# Supplementary material for: Magmatic plumbing and dynamic evolution of the 2021 La Palma eruption
Source: Nat Commun. 2023 Jan 23;14:358. doi: 10.1038/s41467-023-35953-y (PMC9870893; doi:10.1038/s41467-023-35953-y)
Supplement: Supplementary file 1 — Supplementary Material [file 41467_2023_35953_MOESM1_ESM.pdf]

Supplementary Material

Magmatic plumbing and dynamic evolution of the 2021 La Palma eruption

Carmen del Fresno[1,*], Simone Cesca[2], Andreas Klügel[3], Itahiza Domínguez Cerdeña[4], Eduardo A. Díaz-Suárez[4], Torsten Dahm[2,5], Laura García-Cañada[1], Stavros Meletlidis[4], Claus Milkereit[2], Carla Valenzuela-Malebrán[2,5], Rubén López-Díaz[1], Carmen López[1]

[1] Instituto Geográfico Nacional (IGN), Madrid, Spain

[2] GFZ German Research Centre for Geosciences Potsdam, Germany

[3] Department of Geosciences, University of Bremen, Bremen, Germany

[4] Instituto Geográfico Nacional (IGN), Santa Cruz de Tenerife, Spain

[5] Institute of Geosciences, University of Potsdam, Potsdam-Golm, Germany

*Corresponding author: Dr. Carmen del Fresno, Instituto Geográfico Nacional (IGN), Madrid, Spain, cdelfresno@mitma.es

This document provides supplementary information in support of the results and discussion in the main manuscript. The material consists of 8 Supplementary Notes, 14 Supplementary Figures and Supplementary References.

Supplementary Note 1. Data overview

Seismic and deformation data used in this work have been obtained from the permanent Volcano Monitoring Network of the IGN[1], the temporary seismic network installed by IGN and GFZ[2] during the eruption and GRAFCAN network (https://www.grafcan.es/red-de-estaciones). Epicentral distribution of the stations is illustrated in Supplementary Fig 1.

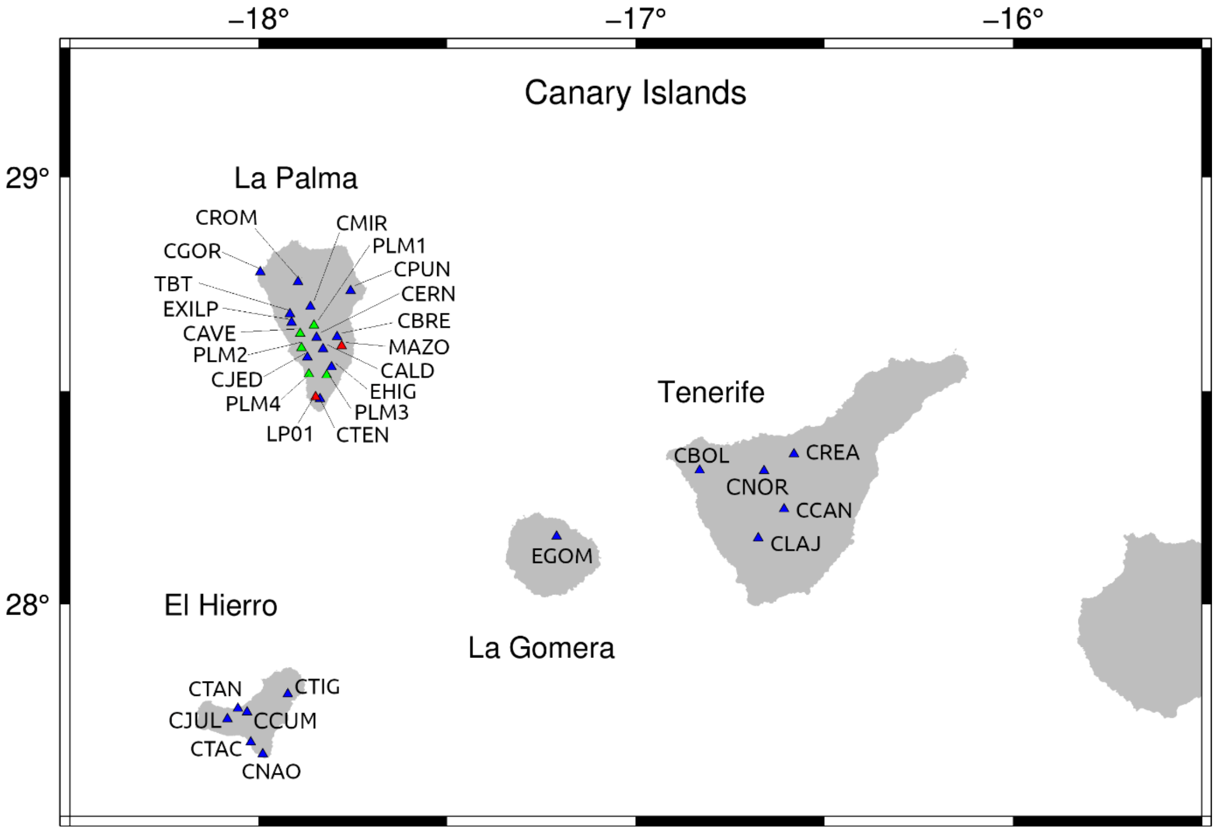

Supplementary Fig. 1: Overview of seismological and geodetic stations used in this study. Blue triangles correspond to permanent seismic stations, green triangles are temporary seismic stations and red squares permanent GNSS stations.

Supplementary Note 2. Velocity model

Hypocentral relocation and moment tensor inversion has been carried out considering the regional velocity model used[3]. The model is illustrated in Supplementary Fig. 2.

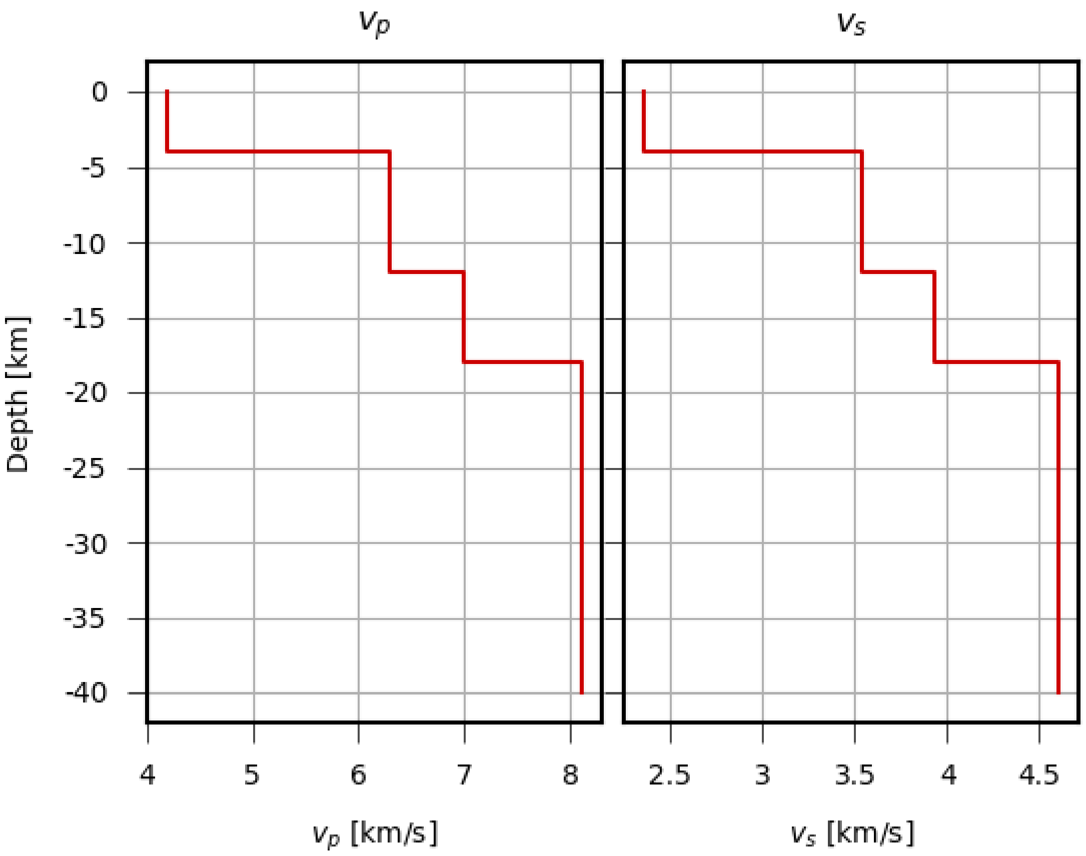

Supplementary Fig. 2: Velocity model used for the hypocentral relocation and moment tensor inversion. The figure shows P and S wave velocity in the uppermost 40 km.

Supplementary Note 3. Seismicity relative relocation.

Results of the relative relocation at different stages of the reactivation are provided as Supplementary Dataset 1 and are illustrated in Supplementary Fig. 3.

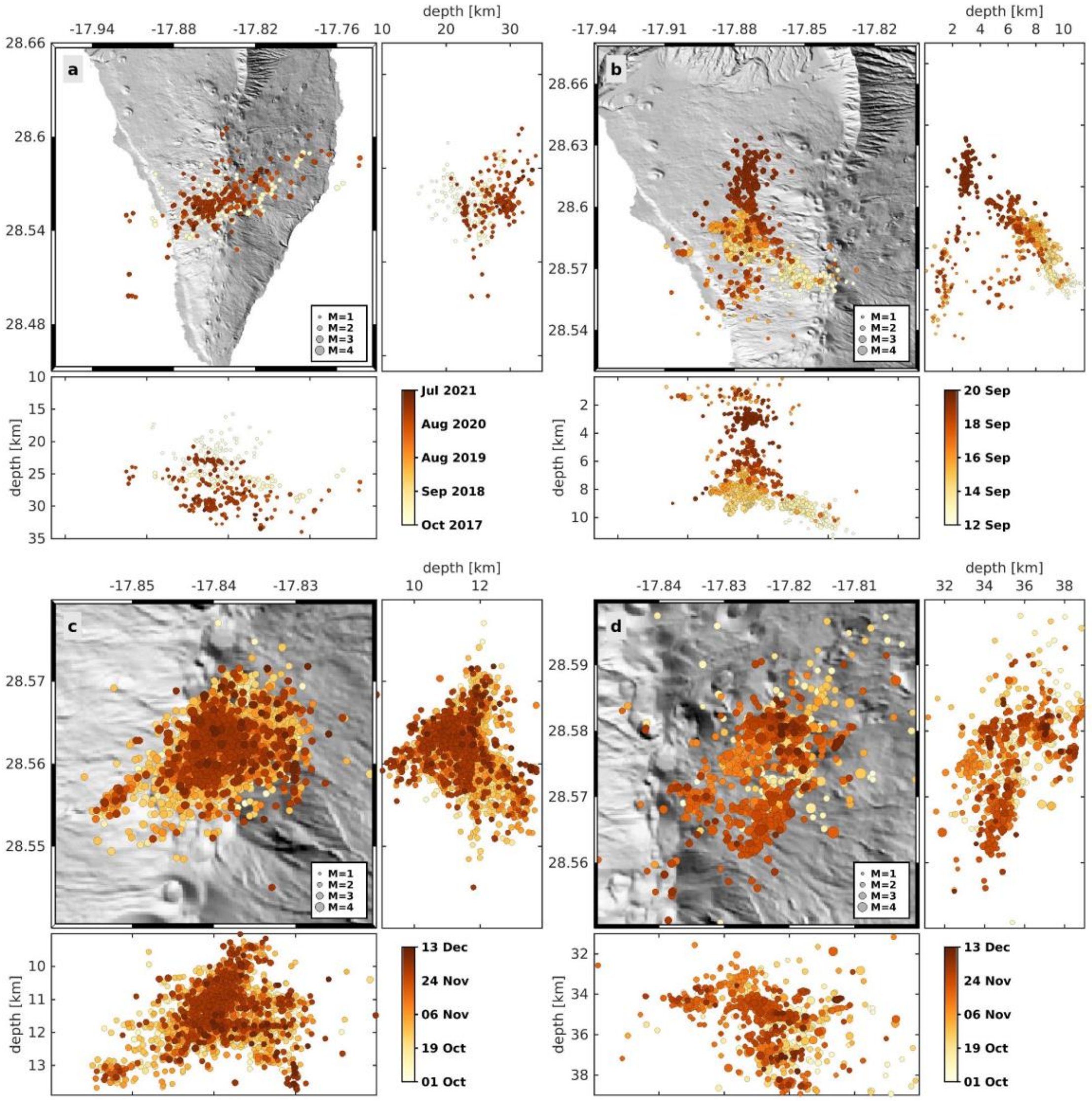

Supplementary Fig. 3: Details of the relocated seismic catalogue using hypoDD algorithm. a. Seismicity of the 2017-2021 swarms before September 11th, 2021 **b.** Pre-eruptive dike related seismicity (11-19 September 2021) **c.** Co-eruptive shallow cluster **d.** Co-eruptive deep cluster.

Supplementary Note 4. Moment Tensor Inversion.

An example of Moment Tensor (MT) solution of an earthquake of the shallow cluster is shown in Supplementary Fig. 4 and a similar illustration of an example of a deep earthquake in Supplementary Fig. 5. The corresponding MT catalogues obtained in this work are provided in Supplementary Datasets 2-3. Clarification of MT clustering results are illustrated in Supplementary Fig. 6 (shallow cluster) and Supplementary Fig. 7 (deep cluster).

Supplementary Fig. 8 shows the relation between local magnitude (M_L) and moment magnitude (Mw) for those earthquakes, for which Mw was estimated by moment tensor inversion.

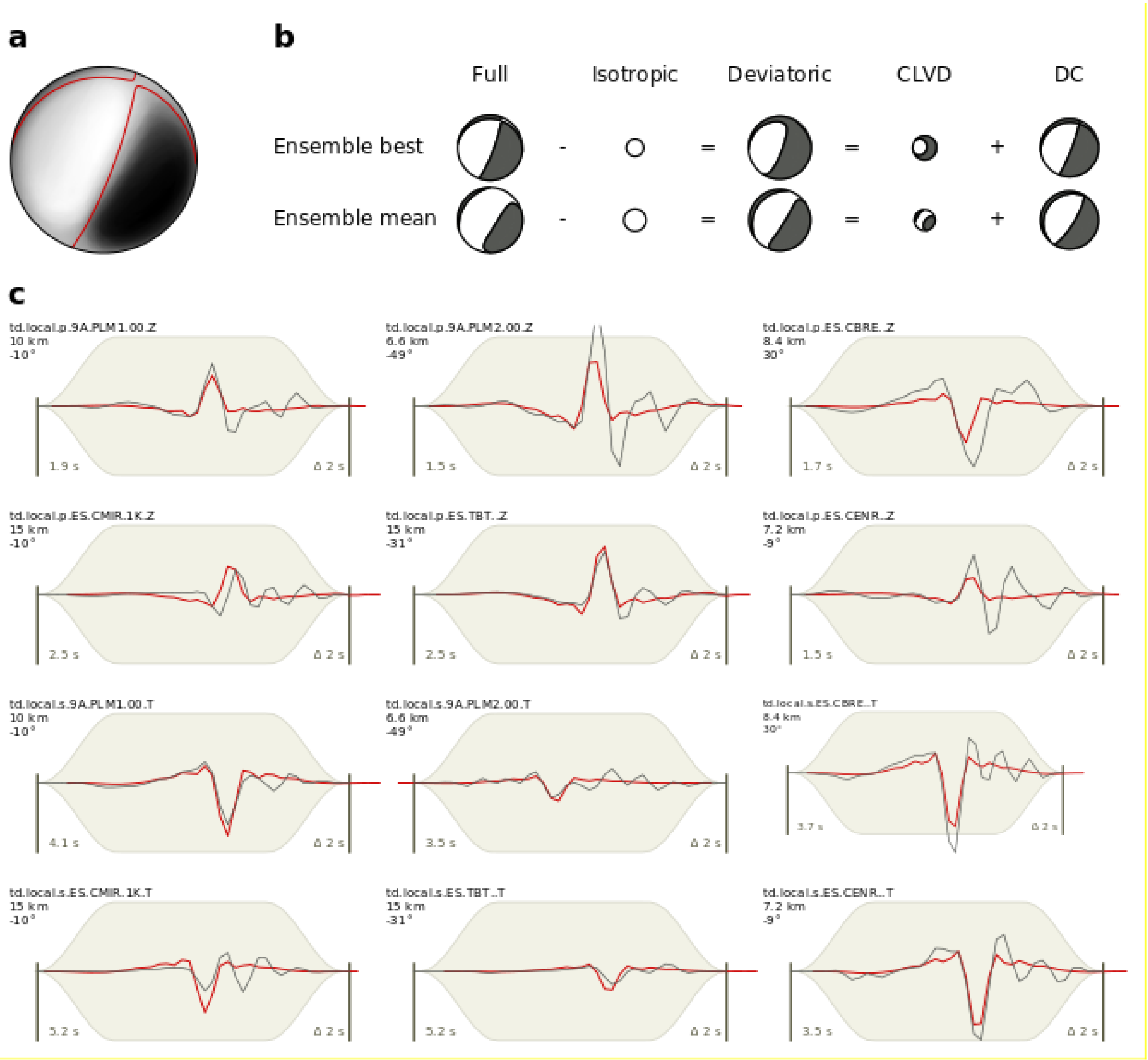

Supplementary Fig. 4: Example of MT result for a shallow earthquake (2021-12-02 05:14, Mw 3.6). a, Overlay of the ensemble of acceptable moment tensor solutions out of the bootstrap analysis (black opaque focal spheres) and best solutions using all data (red lines). **b,** Moment tensor decomposition of the best moment tensor solution, using all data, and the mean solution out of the ensemble of acceptable solutions. **c,** Comparison of observed (black) and synthetic (red) displacement waveforms at selected stations for the P (vertical component) and S wave (horizontal component). The grey areas denote the shape of the taper applied. Next to each panel, station name and component, epicentral distance and azimuth are reported.

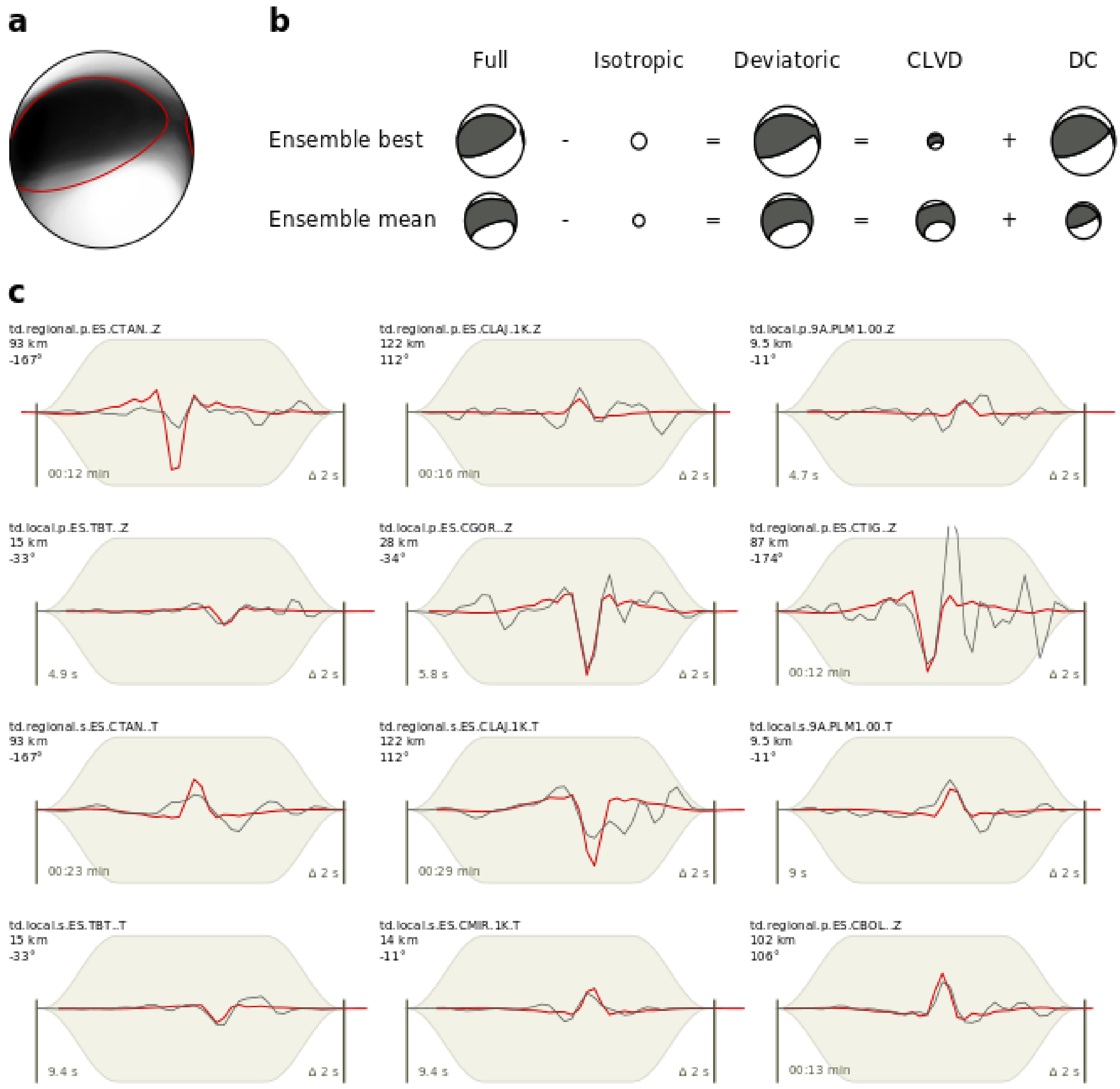

Supplementary Fig. 5: Example of MT result for a deep earthquake (2021-11-03 07:29, Mw 4.1). a, Overlay of the ensemble of acceptable moment tensor solutions out of the bootstrap analysis (black opaque focal spheres) and best solutions using all data (red lines). **b,** Moment tensor decomposition of the best moment tensor solution, using all data, and the mean solution out of the ensemble of acceptable solutions. **c,** Comparison of observed (black) and synthetic (red) displacement waveforms at selected stations for the P (vertical component) and S wave (horizontal component). The grey areas denote the shape of the taper applied. Next to each panel, station name and component, epicentral distance and azimuth are reported.

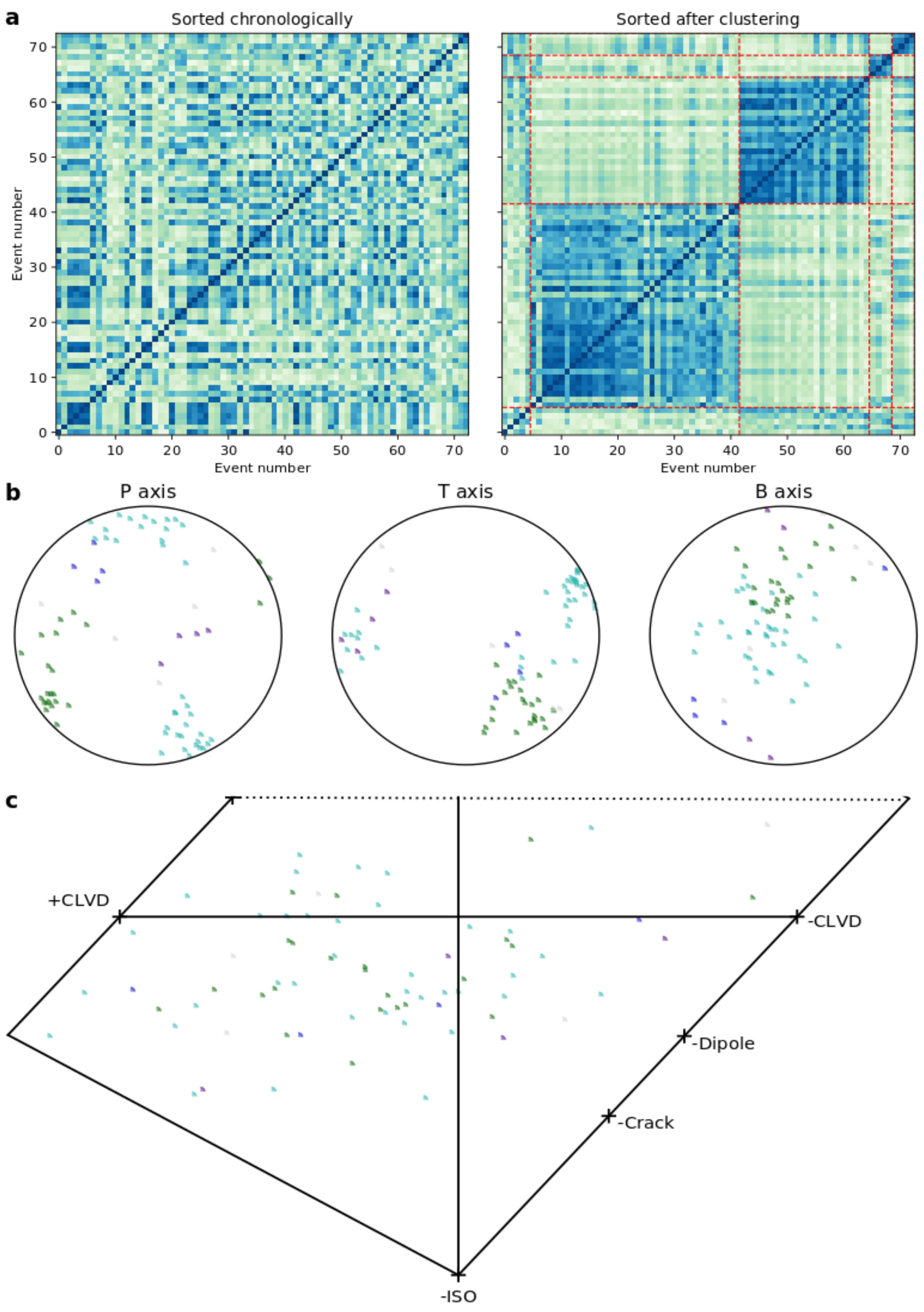

Supplementary Fig. 6: MT clustering results at the shallow cluster. a, Similarity matrix sorted chronologically and after clustering (the white to blue colour scale denotes progressively similar double couple focal mechanisms). **b,** Orientation of the pressure (P), tension (T) and null (B) axis for moment tensors (cluster colours correspond to Fig. 4c, d). **c,** Decomposition of the moment tensor in a Hudson plot (cluster colours correspond to Fig. 4c, d).

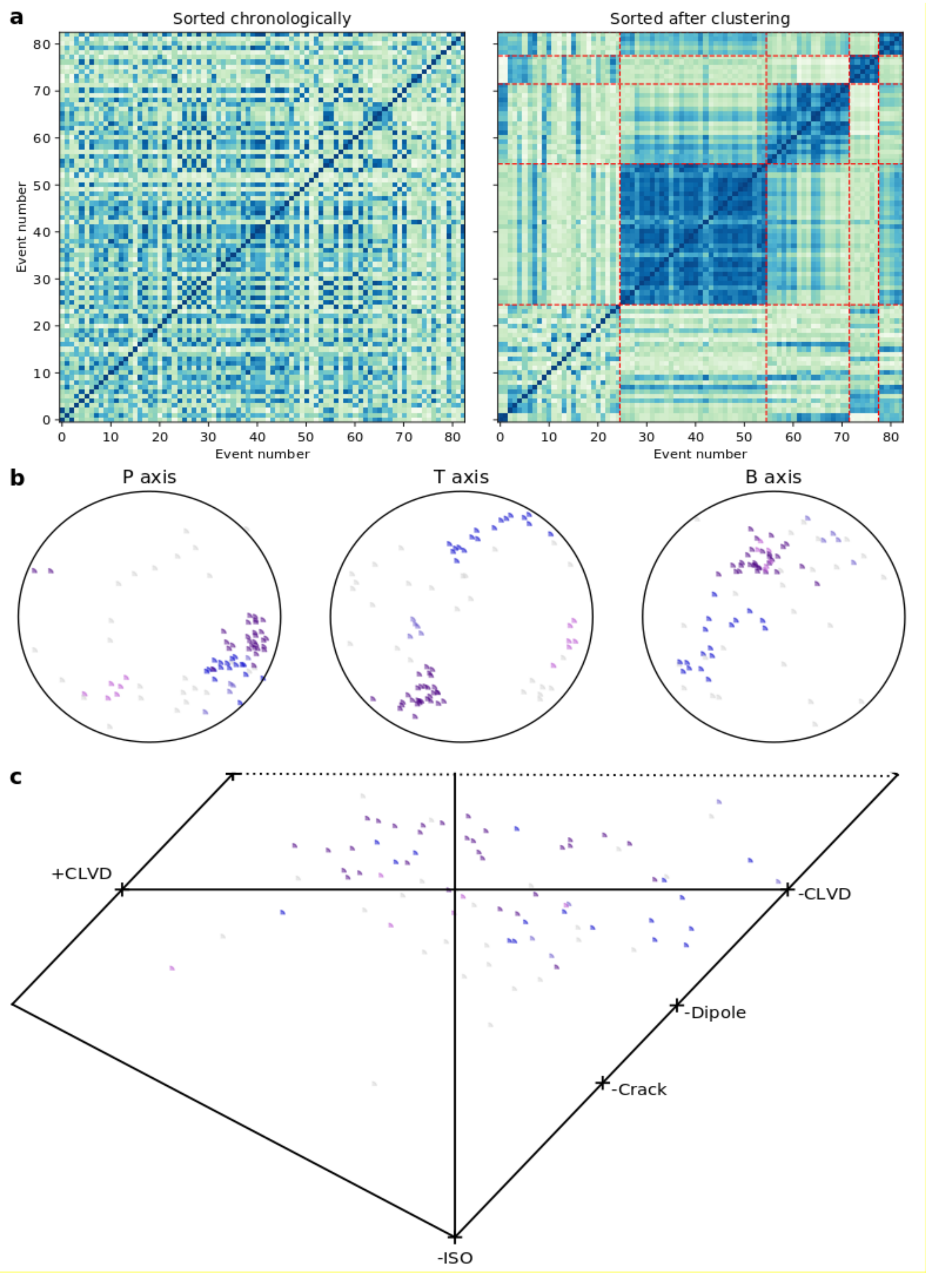

Supplementary Fig. 7: MT clustering results at the deep cluster. a, Similarity matrix sorted chronologically and after clustering (the white to blue colour scale denotes progressively similar double couple focal mechanisms). **b,** Orientation of the pressure (P), tension (T) and null (B) axis for moment tensors (cluster colours correspond to Fig. 5c, d). **c,** Decomposition of the moment tensor in a Hudson plot (cluster colours correspond to Fig. 5c, d).

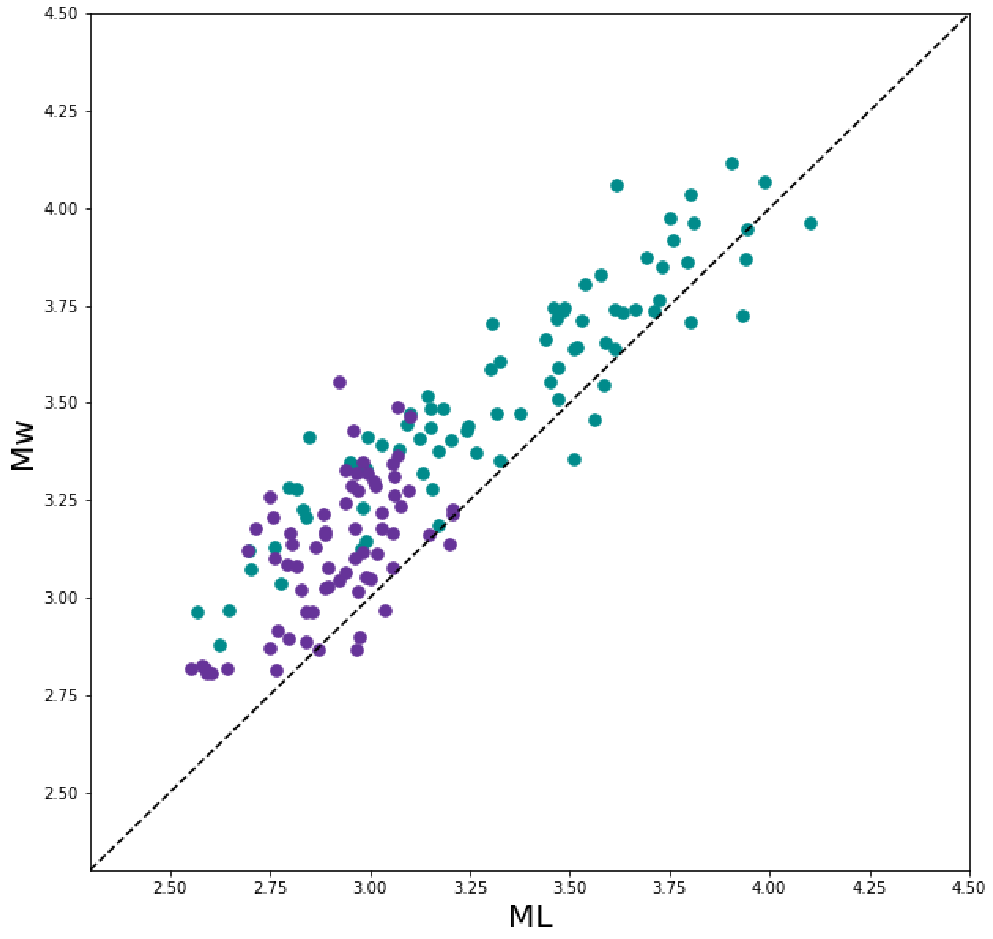

Supplementary Fig. 8: Relation between local magnitude (M_L) and moment magnitude (Mw). Dark violet and green circles correspond to shallow and deep earthquakes, respectively. A dashed line marks a relation Mw = M_L.

Supplementary Note 5. Spatial clustering and waveform similarity.

The relocated earthquakes have been classified into families based on their full waveform cross-correlation. Results for the shallow and deep clusters are shown in Supplementary Fig. 9 and Supplementary Fig. 10, respectively. In addition, some examples of velocity waveforms of the most populated families in each cluster are illustrated in Supplementary Fig 11.

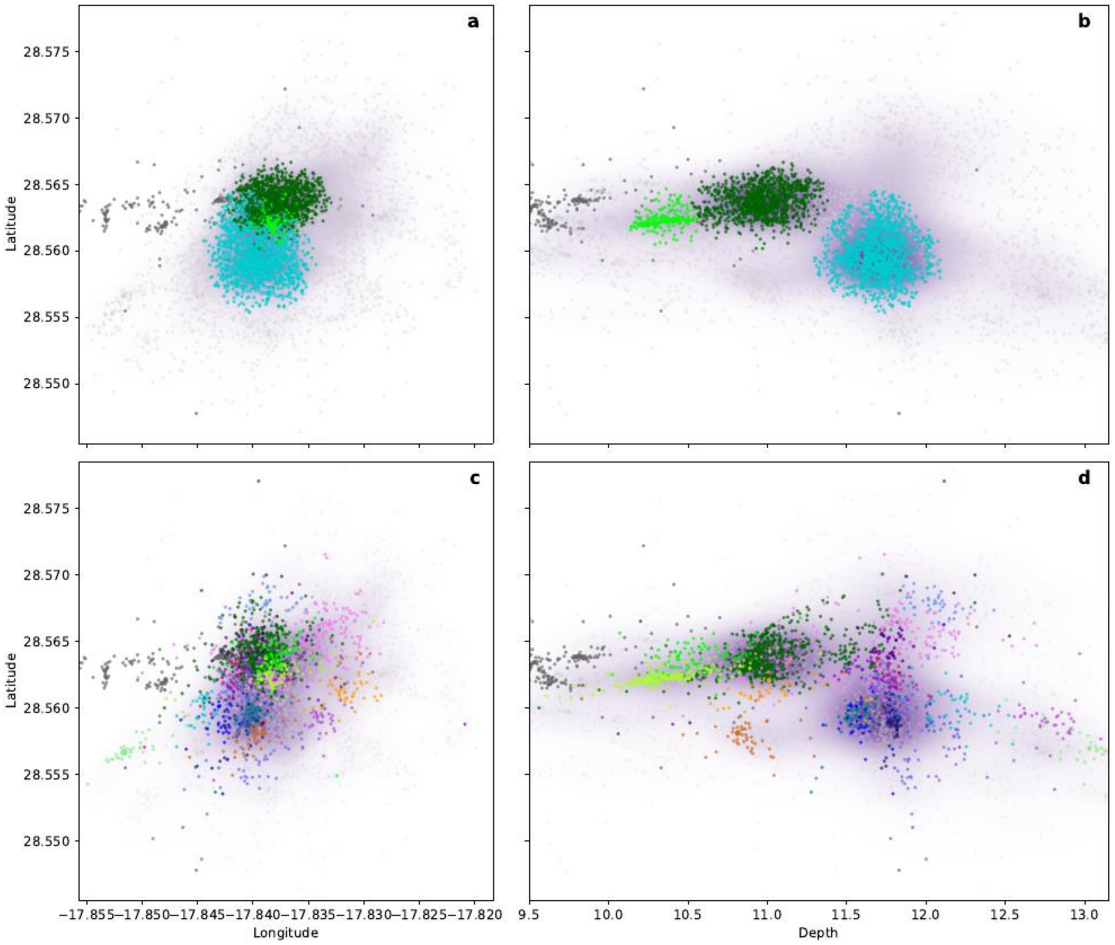

Supplementary Fig. 9: Results of spatial clustering and waveform similarity at the shallow cluster. a, Spatial clustering (colored dots) and hypocentral density (purple, darker regions corresponding to higher density) of relocated hypocentres at the shallow cluster in map view and **b,** along a NS cross section. **c,** Classification based on the waveform similarity (20 families identified by different color dots) and hypocentral density (purple) in map view and **d,** NS cross section (as in panel b). Grey dark dots denote events associated to the dike intrusion, and light grey dots unclustered/unclassified earthquakes.

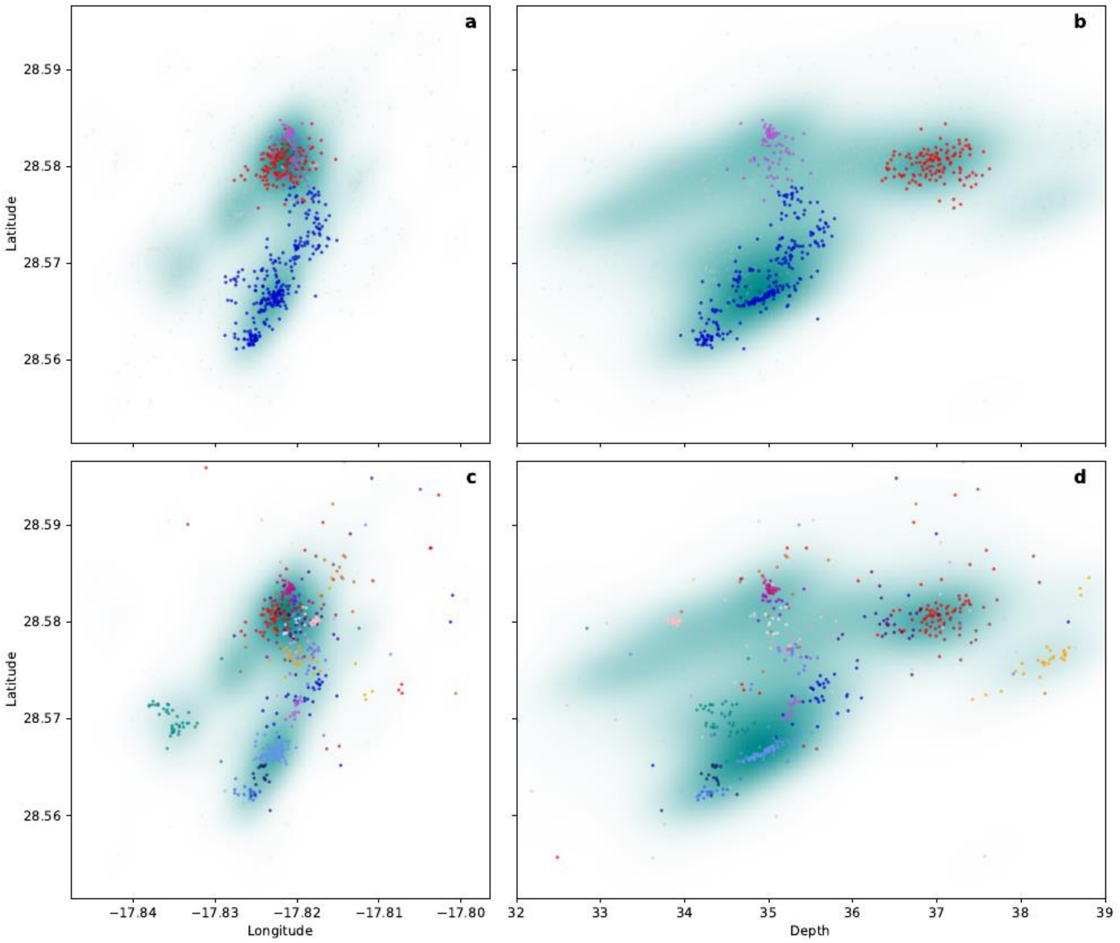

Supplementary Fig. 10: Results of spatial clustering and waveform similarity at the deep cluster. a, Spatial clustering (colored dots) and hypocentral density (dark cyan, darker regions corresponding to higher density) of relocated hypocentres at the deep cluster in map view and **b,** along a NS cross section. **c,** Classification based on the waveform similarity (20 families identified by different colour dots) and hypocentral density (dark cyan) in map view and **d,** NS cross section (as in panel b). Light grey dots denote unclustered/unclassified earthquakes.

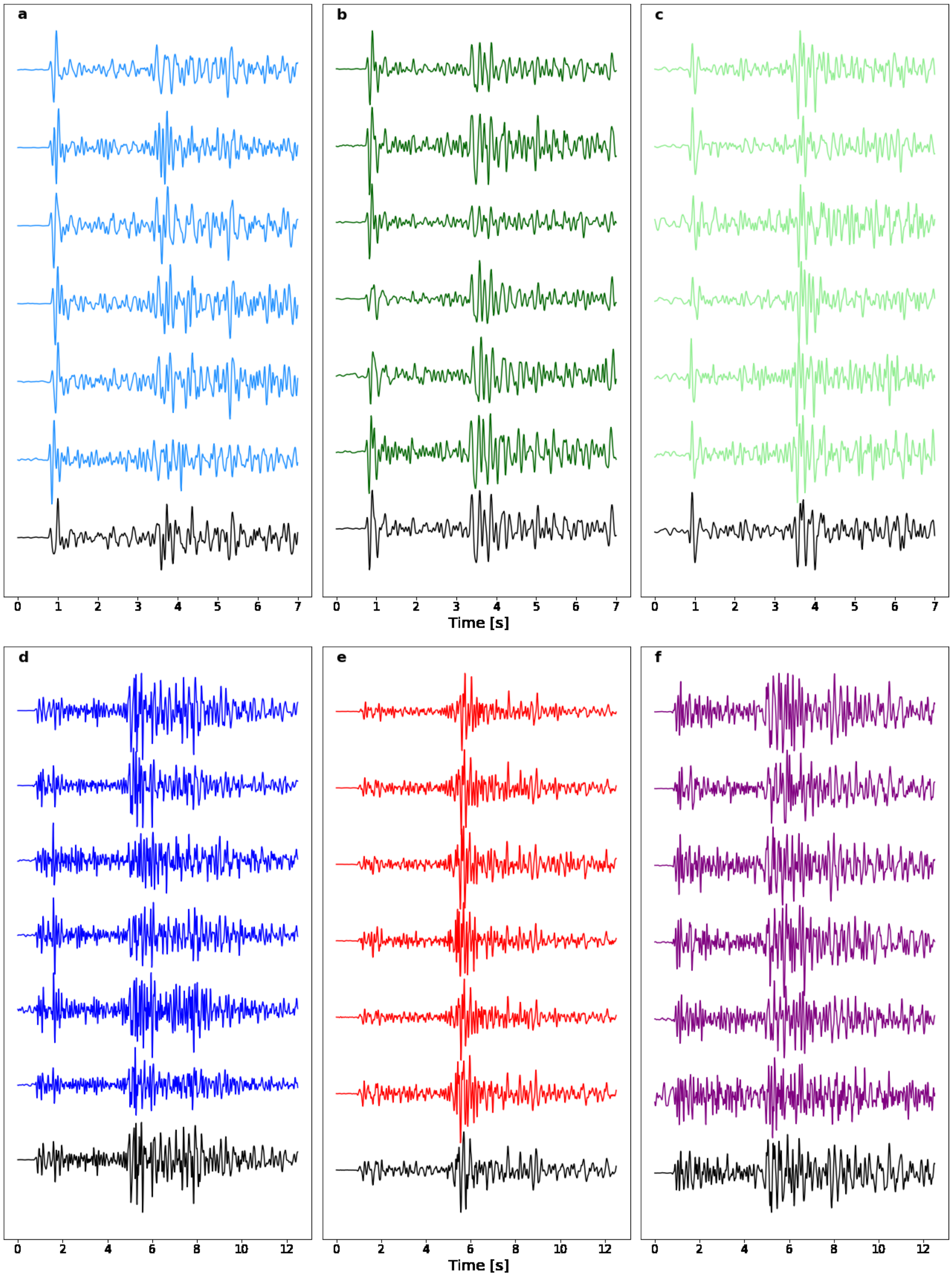

Supplementary Fig. 11: Examples of velocity waveforms. Some earthquakes belonging to the most populated families obtained by means of waveform similarity at the shallow cluster **(a, b, c)** and at the deep cluster **(d, e, f)**, recorded at the TBT station, vertical component. Colours correspond to those used in Fig. 4 and Fig. 5 respectively. Black traces at the bottom correspond to stacked waveforms of each family.

Supplementary Note 6. Stress inversion

Results of the stress inversion using a least squares optimization method are illustrated in Supplementary Fig. 12.

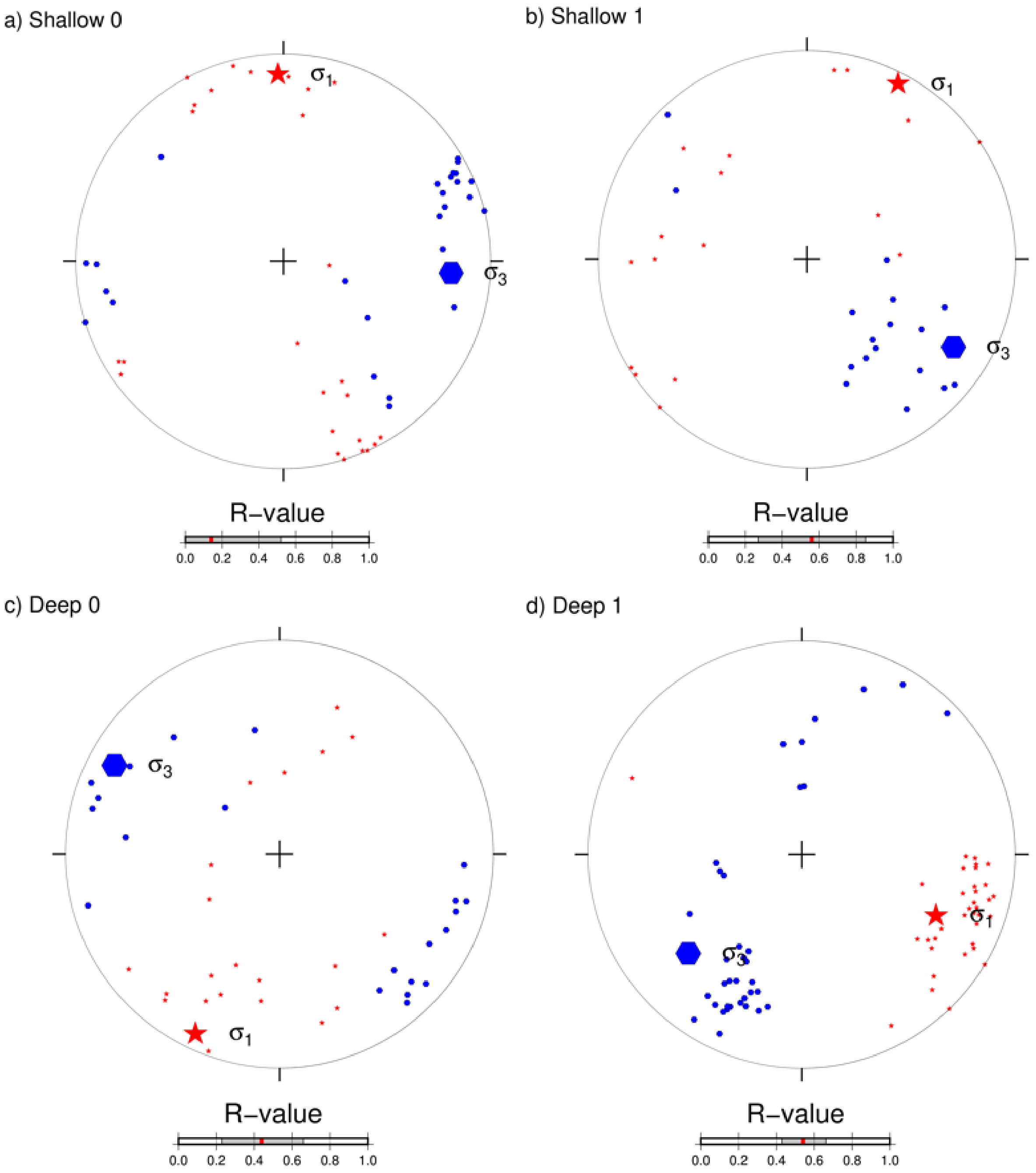

Supplementary Fig. 12: Stress inversion results. Results are shown for two families of the shallow cluster (**a, b**) and two families of the deep one (**c, d**). σ_1 (red star) and σ_3 (blue hexagon) indicate maximal and minimal compression, respectively. Small dots

show projections of P (red) and T axis (blue) of the moment tensor cluster used as input to the inversion (see Fig. 4c and 5c). R-values and its uncertainties are plotted in the bottom bar. They indicate the shape of the stress tensor and are defined by R= $(\sigma_1 - \sigma_2) / (\sigma_1 - \sigma_3)$, where σ_2 is the magnitude of the intermediate principal stress. The inversion method assumes that stress is homogeneous in the volume of the selected subcluster and that slip on each unfavourable oriented fault is in the direction of maximal strain energy release. The total energy release maximum is found by least squares method[4,5].

Supplementary Note 7. Stress modelling

We have developed stress models that can roughly explain some of our observations:

A pressurized reservoir can explain the migration path of the eruptive dikes before the eruption (Supplementary Fig.13a) and a depleted reservoir accounts for the stress rotations and the seismicity distribution within the clusters (Supplementary Fig. 13b).

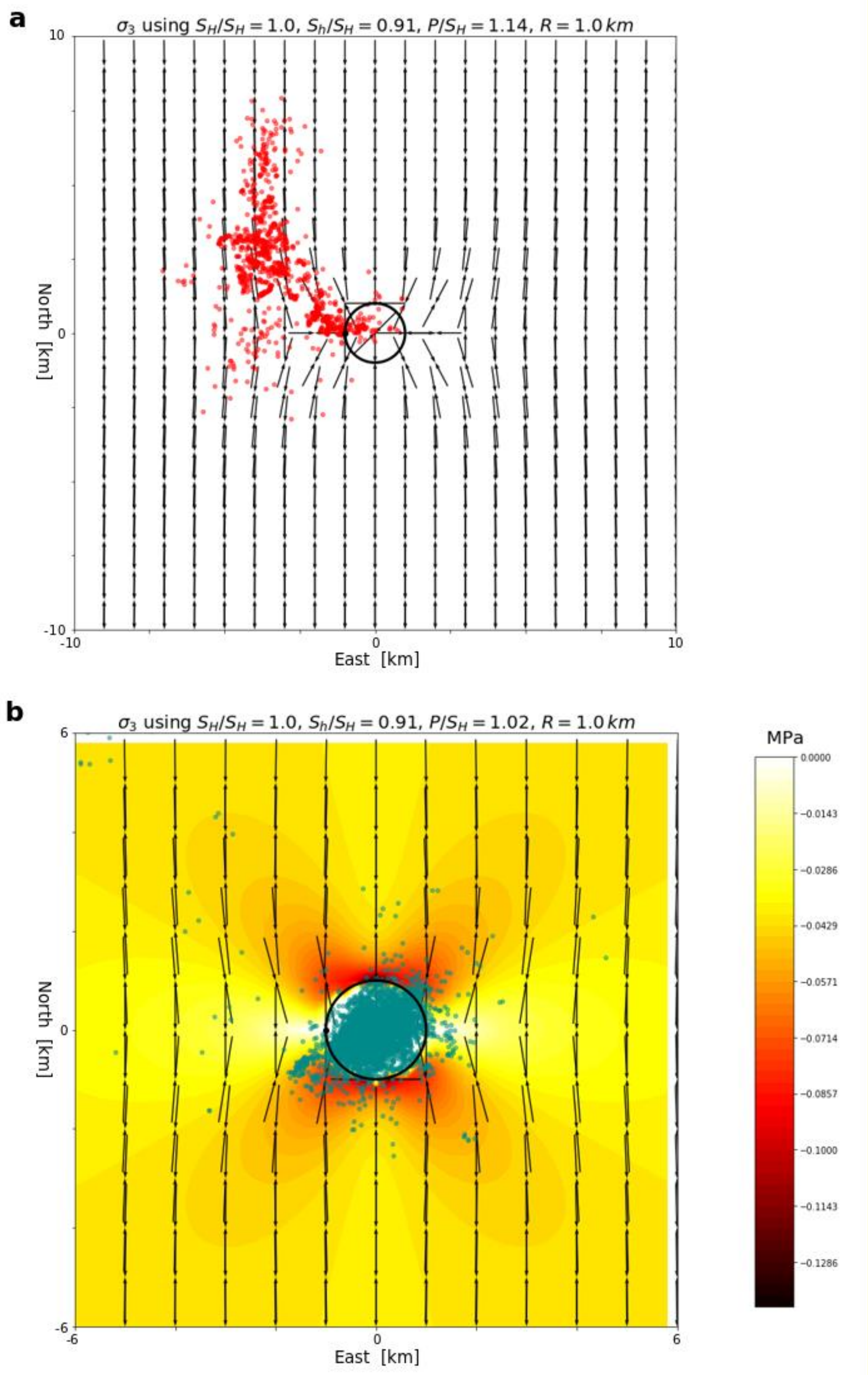

Supplementary Fig. 13: Stress modelling. a, pressurised (P/SH=1.14) and **b,** depleted (P/SH=1) reservoir (large circle, radius R). SH, Sh and P define the remote maximal and minimal compressive stress and the pore pressure in the reservoir, respectively.

Analytical solutions (2D) were taken from Kirsch (1898)[6], Jäger and Cook (1979)[7]. Double arrows indicate the direction of the maximal compressive stress (σ_1). The red circles in (a) show the trace of earthquakes induced by the dike that formed between the shallow reservoir and fissure eruption at the surface (see red points in Fig. 2). We defined the orientation of the maximum compression far from the circular reservoir by the trace of dike induced seismicity. The curved path of the dike between the circular reservoir and the surface follows the direction of maximal compressive stress, as predicted by pressurized reservoir model in (a). The colored grid in (b) shows the magnitude of the maximal shear stress component. Cyan circles show the seismicity of the shallow reservoir cluster. Earthquakes cluster in regions of enhanced shear stress of the depleting reservoir model.

Supplementary Note 8. GNSS velocity results

Vertical and horizontal velocities have been computed from daily coordinates time series (Fig. 3f-h) using a simple linear regression method during the eruptive period. Results are illustrated in Supplementary Fig. 14 together with the relocated co-eruptive seismic clusters.

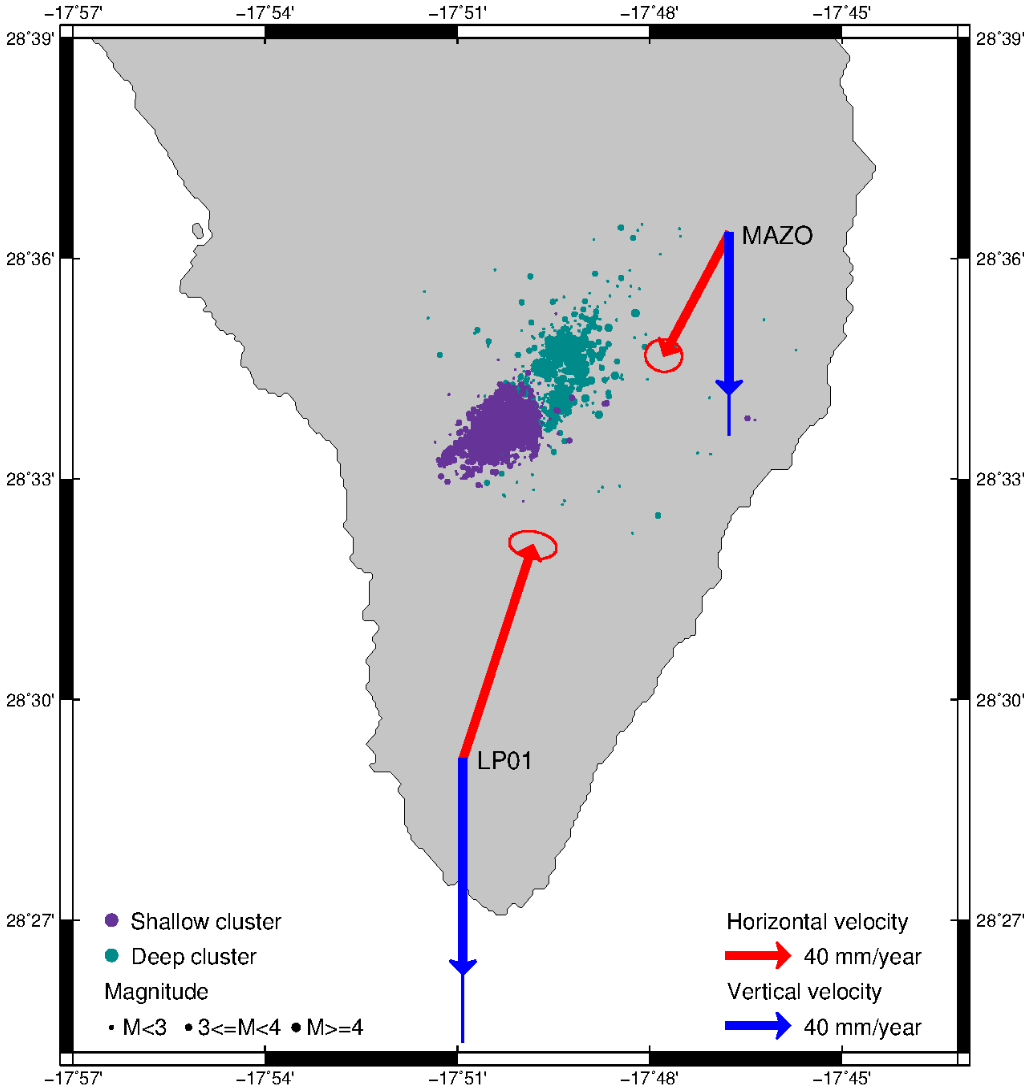

Supplementary Fig. 14: GNSS velocity results computed from 20 September to 13 December 2021. Horizontal (red arrows) and vertical (blue arrow) yearly velocities and their corresponding errors (red ellipse and blue line). Dark violet and green circles correspond to relocated seismicity of the shallow and deep clusters respectively. Size of the circle according to the earthquake magnitude.

Supplementary References

1. Instituto Geografico Nacional. Spanish Digital Seismic Network [Data set]. International Federation of Digital Seismograph Networks. https://doi.org/10.7914/SN/ES (1999)
2. Walter, T. *et al.* HART-La Palma volcanic eruption. GFZ Data Services. Other/Seismic Network. https://doi.org/10.14470/4N7576350874 (2021)
3. Dañobeitia, J. J. Interpretación de la estructura de la corteza en el Archipiélago Canario a partir de perfiles sísmicos profundos de refracción. PhD Thesis. Universidad Complutense de Madrid (1980).
4. Petersen, G. M. *et al.* Regional centroid moment tensor inversion of small to moderate earthquakes in the Alps using the dense AlpArray seismic network: Challenges and seismotectonic insights. *Solid Earth* **12**, 1233–1257 (2021).
5. Cesca, S. *et al.* The Mw 8.1 2014 Iquique, Chile, seismic sequence: A tale of foreshocks and aftershocks. *Geophys. J. Int.* **204**, 1766–1780 (2016).
6. Kirsch, G. Die Theorie der Elastizität und die Bedürfnisse der Festigkeitslehre. *Zeitschrift des Vereines deutscher Ingenieure* vol. 42 (1898).
7. Jaeger, J. C. & Cook, N. G. W. Fundamentals of Rock Mechanics (Chapman and Hall, London, 1971).
