# Supplementary material for: Magmatic plumbing and dynamic evolution of the 2021 La Palma eruption
Source: Nat Commun. 2023 Jan 23;14:358. doi: 10.1038/s41467-023-35953-y (PMC9870893; doi:10.1038/s41467-023-35953-y)
Supplement: Supplementary file 2 — Description of Additional Supplementary files [file 41467_2023_35953_MOESM2_ESM.docx]

**Seismic catalogues:**

● Supplementary Dataset 1: Relocated catalogue, counting 8,488 events from 11.9.2021 to 31.12.2021. (file suppldataset1.txt)

● Supplementary Dataset 2: Catalogue of moment tensors at the shallow cluster, including 73 events. (file suppldataset2.txt)

● Supplementary Dataset 3: Catalogue of moment tensors at the deep cluster, including 83 events. (file suppldataset1.txt)
